# Itraconazole promotes melanoma cells apoptosis via inhibiting hedgehog signaling pathway-mediated autophagy

**DOI:** 10.3389/fphar.2025.1545243

**Published:** 2025-01-23

**Authors:** Shunqiao Jin, Xiaojiao Liu, Lingqin Cai, Jiayu Yan, Ling Li, Hongjun Dong, Yuxue Gao, Xicong Zhu, Cong Zhang, Xuezhu Xu

**Affiliations:** ^1^ Department of Dermatology, Second Affiliated Hospital of Dalian Medical University, Dalian, China; ^2^ Department of Dermatology, Taizhou Hospital of Zhejiang Province Affiliated to Wenzhou Medical University, Linhai, China; ^3^ Department of Dermatology, Chengdu Badachu Medical Aesthetics Hospital, Chengdu, China; ^4^ Department of Dermatology, Taizhou Rehabilitation Hospital, Taizhou Enze Medical Center (Group), Taizhou, China; ^5^ Department of Dermatology, The Affiliated Zhongshan Hospital of Dalian University, Dalian, China; ^6^ Department of Preventive Medicine, Dalian Medical University, Dalian, China

**Keywords:** itraconazole, melanoma, autophagy, hedgehog pathway, apoptosis, machine learning

## Abstract

**Background:**

Itraconazole, a widely used antifungal medication, has shown potential in inhibiting tumor growth and reducing angiogenesis. However, its role in melanoma tumor growth remains insufficiently explored. This study investigates the inductive effect of itraconazole on autophagy-mediated apoptosis in melanoma cells.

**Method:**

Potential drug targets were identified using the PMF machine learning algorithm. Apoptosis and cell cycle in melanoma cell lines A375 and A2058 were assessed via flow cytometry. Western blot analysis was performed to examine autophagy and associated signaling proteins, while autophagy flux and autophagosome formation were visualized using fluorescence microscopy. A melanoma cell xenograft mouse model was established to evaluate the inhibitory mechanisms of itraconazole on tumor cell proliferation.

**Result:**

Using the PMF machine learning algorithm, SQSTM1 was identified as the primary target of itraconazole. Itraconazole inhibited melanoma cell proliferation by inducing G1 phase arrest and autophagy-mediated apoptosis in A375 and A2058 cells. Furthermore, itraconazole suppressed Hedgehog signaling and counteracted the activation of the Hedgehog agonist recombinant human Sonic Hedgehog (rhShh). *In vivo*, itraconazole significantly reduced tumor growth in A375 and A2058 xenograft models.

**Conclusion:**

Itraconazole induces autophagy-mediated apoptosis in melanoma cells by inhibiting Hedgehog signaling, underscoring its potential as a therapeutic option for melanoma treatment.

## 1 Introduction

Melanoma is a melanocytes-derived malignant tumor (Lo and Fisher, 2014). Globally, Melanoma constitutes about 1.7% of all newly identified primary malignant tumors and roughly 0.7% of all cancer-related fatalities (Schadendorf et al., 2018). The primary treatments for melanoma include surgical intervention, radiotherapy, and systemic drug therapies (Long et al., 2023). Despite significant reductions in postsurgical recurrence, many patients experience non-responsiveness, treatment resistance, or severe toxicities (Huppert and Daud, 2022). Itraconazole, a triazole class antifungal drug, inhibits ergosterol synthesis in fungal cell membranes, thereby preventing fungal growth (de Macedo-Silva et al., 2013). However, growing evidence suggests that itraconazole not only possesses antifungal properties but also exerts antitumor effects on various cancer cells, such as basal cell carcinoma (BCC), and non-small lung cancer (NSLC) (Aftab et al., 2011; Ally et al., 2016). A case report indicated that a patient suffering vagina primary malignant melanoma treated with itraconazole for 30 days showed a reduction in tumor size under PET-CT scans taken on days 6 and 30 (Inoue et al., 2018). Itraconazole exerts its anticancer activity through multiple mechanisms, such as inhibiting angiogenesis, blocking the Hedgehog signaling pathway, and disrupting cholesterol biosynthesis (Pounds et al., 2017), all of which are critical for tumor growth and metastasis. In melanoma, the aberrant activation of the Hedgehog pathway plays a pivotal role in tumor progression (Li et al., 2011), making it an attractive target for Itraconazole. The underlying molecular mechanisms of Itraconazole in melanoma remains under investigation, with further research needed to establish its therapeutic potential.

Autophagy is a catabolic process that involves various autophagy-related (ATG) gene products that coordinate the way double-membrane vesicles are formed, called autophagosomes (Nishimura and Tooze, 2020). These vesicles sequester and reduce damaged proteins and organelles in lysosomes, recycling cellular components and energy to guarantee sustainable cellular homeostasis to deal with stress, such as those in the tumor microenvironment (Mizushima and Komatsu, 2011; Kocaturk et al., 2019). Autophagy can either facilitate cancer development or inhibit tumor progression, considering different disease stages and tumor microenvironments (Chung et al., 2020). According to recent studies, itraconazole is capable of triggering autophagy in glioblastoma and breast cancer cells, so as to prevent cancer being developed (Liu et al., 2014; Wang et al., 2017). Additionally, itraconazole induces cell death with autophagy and suppresses transketolase expression, adding its effectiveness in treating colon cancer and increasing patient survival rates (Shen et al., 2021). Literature reports suggest that itraconazole inhibits tumors through autophagy, and exploring its mechanism is becoming increasingly necessary. Itraconazole suppresses pancreatic cancer via the TGF-β/SMAD2/3 signaling pathway (Chen et al., 2018). It exerts anti-hepatocellular carcinoma effects by virtue of ROS, Wnt/β-catenin, Hedgehog (Hh), and AKT/mTOR/S6K pathways (Wang et al., 2020).

The Hedgehog (Hh) signaling pathway is frequently dysregulated in melanoma, contributing to tumor growth and metastasis (Li et al., 2011). The Hh signaling pathway crucially affects tissue homeostasis and embryonic development (Warner and McClay, 2014),which sees the binding of the Sonic Hedgehog (SHH) ligand binds to the Patched Homolog 1 (PTCH1) receptor protein, promoting the Smoothened (SMO) prote into be released, which triggers an intracellular cascade that regulates various cellular functions (Peng et al., 2022). Abnormal Hh signaling is linked to tumorigenesis and malignancy in several cancers (Jiang, 2022), including prostate, breast, pancreatic, hepatocellular, and gastric cancers (Karhadkar et al., 2004; Riobo-Del Galdo et al., 2019; Thayer et al., 2003; Huang et al., 2006; Berman et al., 2003). SHH signaling has been shown to repress autophagy in stromal stellate cells, thereby reducing autophagy-induced alanine secretion to achieve the inhibition of tumor growth (Sousa et al., 2016). Conversely, GANT61, a Gli1 inhibitor, induces autophagy (Sun et al., 2023). In the study by Pan et al. SHH signaling pathway adjusts ovarian cancer cell autophagy and migration (Pan et al., 2021). Hu et al. found the role of itraconazole in inducing apoptosis and cell cycle arrest while restricting the Hh signaling pathway (Hu et al., 2017). Itraconazole prevents BCC growth and division by inhibiting the SMO protein in the Hh signaling pathway (Kim et al., 2014). According to previous studies, itraconazole might restrain cancer cell growth via deactivating the Hh signaling pathway. Therefore, in our study, we investigate whether itraconazole regulates autophagy in melanoma through its effects on the Hh signaling pathway.

## 2 Materials and methods

### 2.1 Melanoma cell culture techniques

The melanoma cell line A375 was obtained from the Cell Bank/Stem Cell Bank of the Chinese Academy of Sciences, while A2058 and SK-MEL-28 cell lines were purchased from Shanghai Yihe Applied Biotechnology Corporation. SK-MEL-28 cells were cultured in RPMI 1640 medium (Gibco, United States) supplemented with 10% fetal bovine serum (FBS) (Biological Industries, Israel). A2058 and A375 cells were cultured in DMEM (Gibco, United States) supplemented with 10% FBS. All cell lines were incubated at 37°C in a humidified atmosphere containing 5% CO_2_.

### 2.2 Proliferation assay

SK-MEL-28, A375, and A2058 cells were treated with itraconazole at varying concentrations for 48 h. After treatment, 10 µL of CCK-8 solution (#C6005, New Cell and Molecular Biotechnology, China) was added to each well, followed by incubation for 3 h. Cell viability was measured using a microplate reader (Spectra Max M4, United States) at an absorbance of 450 nm. The experiment was conducted in six replicates. The IC50 values were determined, and cell viability was calculated as the ratio of the optical density (OD) value of the itraconazole-treated group to that of the control group. Based on IC50 values, A2058 and A375 cells were selected for further experiments.

### 2.3 Western blotting

RIPA buffer (#R0020, Solarbio, China) was used to extract total proteins from cells or tissues. Protein concentration was quantified using a Bicinchoninic Acid (BCA) kit (#WB6501, New Cell and Molecular Biotechnology, China). Proteins were separated using SDS-PAGE (8%–15%) and transferred onto PVDF membranes (Millipore, United States). The membranes were blocked for 30 min with a blocking buffer (#P30500, New Cell and Molecular Biotechnology, China), followed by overnight incubation at 4°C with primary antibodies. After washing, the membranes were incubated for 2 h at room temperature with a goat anti-rabbit IgG secondary antibody (#A0208, Beyotime, China). Protein bands were visualized using enhanced chemiluminescence (#P2100, New Cell and Molecular Biotechnology, China). Recombinant human Sonic Hedgehog (rhShh, #Z03067) was obtained from GenScript (Nanjing, China), and the autophagy inhibitor 3-methyladenine (3-MA) was sourced from Selleck Chemicals (Texas, United States).

### 2.4 Colony formation assay

A375 and A2058 cells (1 × 10^3^ cells/dish) were seeded into 35 mm dishes and allowed to adhere. Following adhesion, the cells were treated with itraconazole for 48 h. After the treatment period, itraconazole was removed, and the cells were incubated for an additional 2 weeks to allow visible colony formation. The colonies were fixed in 4% paraformaldehyde (PFA) and subsequently stained with crystal violet. Colonies with a diameter greater than 1 mm (comprising over 50 cells) were counted.

### 2.5 Immunohistochemical analysis

Tumor tissues excised from xenograft tumor mice were fixed, paraffin-embedded, and sectioned at a thickness of 4 μm. The sections were mounted on slides, dewaxed, and incubated overnight at 4°C with a mouse anti-Ki-67 antibody (#28074-1-AP, Proteintech Group, China). Immunostaining intensity was evaluated in at least three independent fields to calculate the average intensity score. The immunostaining level was graded as follows: no staining (score 0), weak staining (score 1), moderate staining (score 2), and strong staining (score 3). The proportion of positively stained cells was classified into five groups: ≤5% (score 0), 6%–25% (score 1), 26%–50% (score 2), 51%–75% (score 3), and >75% (score 4). The total score for each slide was determined by summing the intensity and proportion scores (Birner et al., 2000; Goh et al., 2008).

### 2.6 Transmission electron microscopy observations

After 48 h of itraconazole exposure, A375 and A2058 cells were collected and fixed in 2.5% glutaraldehyde. The cells were rinsed three times with PBS and dehydrated using a graded series of ethanol. Sections were stained with a 2% uranyl acetate-saturated alcohol solution and examined using a transmission electron microscope (TEM, Japan Electronics, Japan). Images were captured at magnifications of 2,000× (2K) and 10,000× (10K).

### 2.7 Fluorescence microscopy observations

A375 and A2058 cells were transfected for 24 h with an adenovirus containing the mCherry-eGFP-LC3B gene (Suzhou Genepharma, China), a marker used for tracking autophagy flux. After transfection, the cells were rinsed with PBS and treated with itraconazole for 48 h. The cells were then fixed with 4% paraformaldehyde (PFA, Jijia Biotechnology, China) for 20 min, followed by another PBS wash. GFP/RFP signals were visualized and captured using a Zeiss Laser Scanning Confocal Microscope (Zeiss, Germany).

### 2.8 *In vivo* xenograft experiment

The experiments were conducted in accordance with the protocols approved by the Animal Ethics Committee of Taizhou Hospital of Zhejiang Province Affiliated to Wenzhou Medical University (Approval No: TZY-2023224). Female BALB/c nude mice (6–8 weeks old, 20 ± 2 g, n = 24) were obtained from Hangzhou Qizhen Experimental Animal Technology (License No: SCXK2022-0005). Each mouse was subcutaneously injected with 2 × 10^6^ A375 or A2058 cells. When tumor volumes reached 30–50 mm^3^, the mice were randomly divided into two groups: itraconazole treatment (50 mg/kg, once daily, n = 12) and vehicle control (normal saline, n = 12). Treatments were administered *in situ* for 14 days, based on previously published protocols (Zhang et al., 2021; Cai et al., 2022). Tumor dimensions were measured every 3 days using the formula: length × width^2^/2. At the end of the 14-day treatment period, the mice were sacrificed, and the xenograft tumors were harvested. After washing with PBS, tumors were weighed and stored at −80°C for subsequent analyses.

### 2.9 Machine learning via PMF

Probabilistic Matrix Factorization (PMF) stands as a widely adopted machine learning technique that has effectively been utilized to forecast interactions between drugs and their targets. The established interactions involving N drugs and M protein targets are encapsulated within the interaction matrix *R*_*N×M*_ where each element is defined as:

$$Rij=\left\{\begin{array}{l} 1,if\;drug\;i\;interact\;with\;target\;j\\ 0,otherwise\; \end{array}\right.$$

### 2.10 Statistical methodology

Statistical analysis was performed using SPSS Statistics, Version 21.0 (Armonk, NY: IBM Corp.), and data were presented as means ± standard deviations (SD). Normality of the data was confirmed prior to analysis. Group comparisons were conducted using one-way ANOVA, with statistical significance set at P < 0.05.

## 3 Results

### 3.1 Itraconazole inhibits melanoma cells proliferation

**FIGURE 1:**
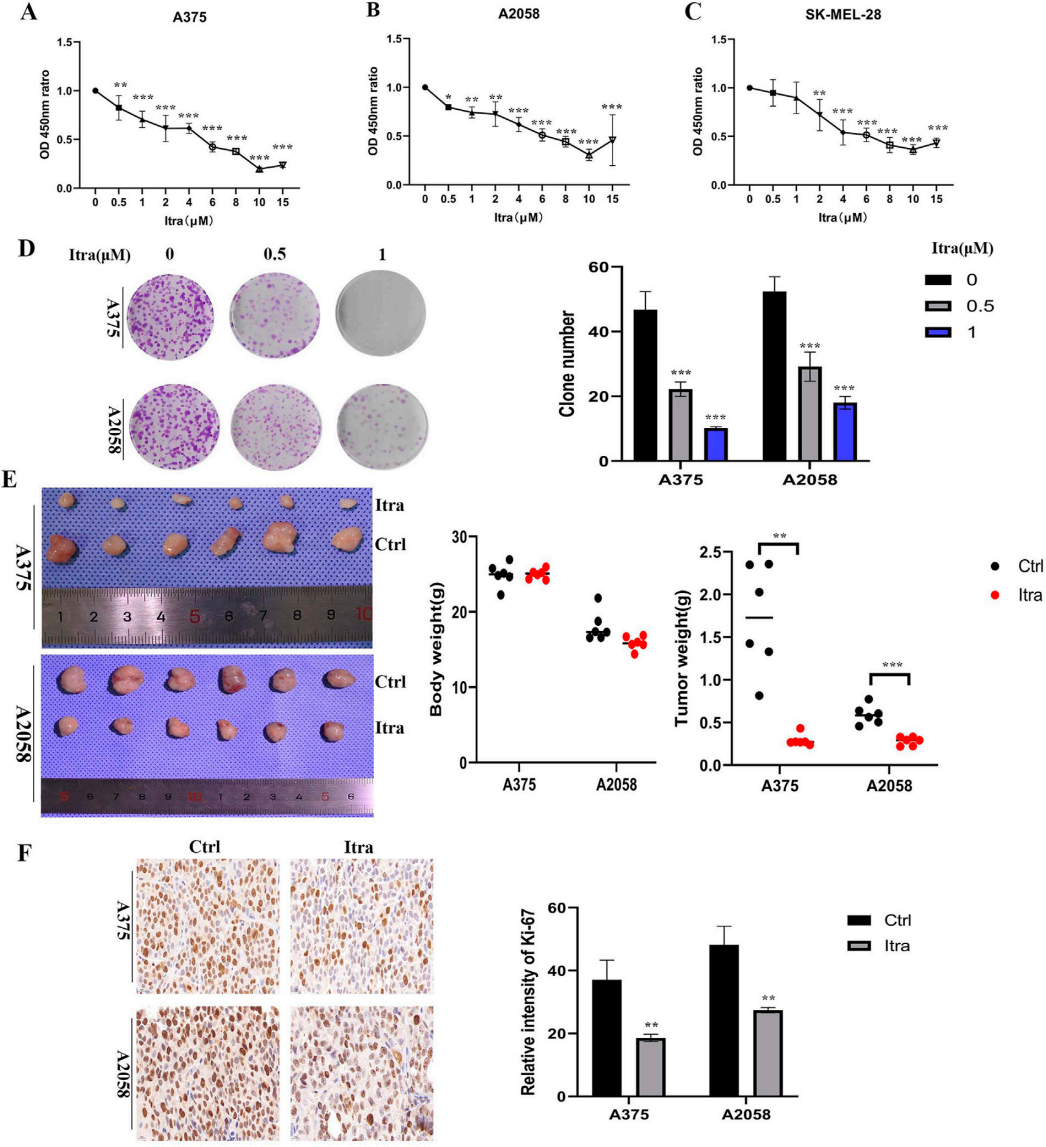
Itraconazole inhibited melanoma cells proliferation *in vitro* and *in vivo*. **(A–C)** Cell viability treated with itraconazole. **(D)** Cell colony formation assay. **(E)** Tumor weight and total weight of the nude mice. **(F)** Ki-67 expression was examined by immunohistochemistry. *P < 0.05; **P < 0.01; ***P < 0.001.

The proliferation rates of A375, A2058, and SK-MEL-28 cells were assessed using CCK8 assays following treatment with itraconazole for 48 h. Cell viability decreased in a concentration-dependent manner across all cell lines (Figures 1A–C). Colony formation assays (Figure 1D) further demonstrated that the itraconazole-treated groups formed significantly fewer colonies, confirming the inhibitory effect of itraconazole on cell proliferation. A375 and A2058 xenografts have different apoptosis and caspase activation patterns (Smith et al., 2012). To evaluate the *in vivo* effects, a subcutaneous xenograft model was established using both A375 and A2058 cells. Nude mice treated with itraconazole showed a significant reduction in tumor growth compared to saline controls (Figure 1E), with no notable differences in body weight between the groups. Immunohistochemistry (IHC) analysis of Ki-67 expression, a marker for tumor cell proliferation, revealed markedly lower Ki-67 levels in itraconazole-treated tumors compared to controls (Figure 1F). These findings underscore the potential of itraconazole as an effective inhibitor of melanoma cell proliferation both *in vitro* and *in vivo*.

### 3.2 Itraconazole induces apoptosis in melanoma cells

**FIGURE 2:**
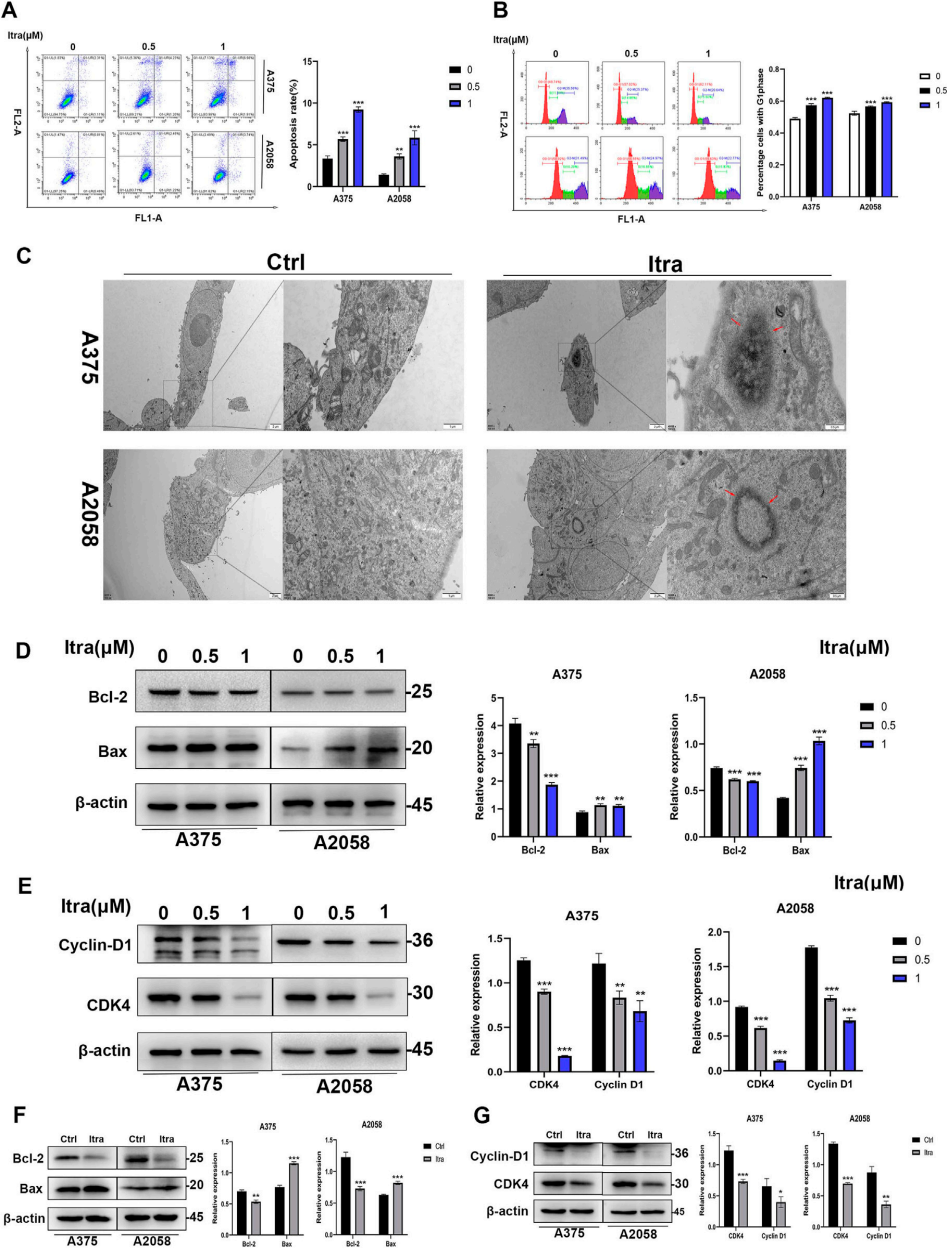
Itraconazole induces apoptosis in melanoma cells. **(A, B)** Cell apoptosis and cycle distributions were examined by flow cytometry. **(C)** Apoptotic bodies were examined by transmission electron microscopy. **(D, E)** Bax, Bcl-2, CDK4 and Cyclin D1 protein level. **(F, G)** Bax, Bcl-2, CDK4 and Cyclin D1 protein level. *P < 0.05; **P < 0.01; ***P < 0.001.

Flow cytometry was used to investigate whether the inhibition of cell proliferation in itraconazole-treated mice was associated with melanoma cell apoptosis. As shown in Figure 2A, a dose-dependent increase in apoptotic cells was observed after 48 h of itraconazole treatment. Transmission electron microscopy (TEM) further confirmed the presence of apoptotic bodies in itraconazole-treated cells (Figure 2C). To explore the potential mechanism of itraconazole’s antiproliferative effects, cell cycle analysis was performed. Figure 2B indicates that itraconazole induced G1 phase arrest in melanoma cells. Western blot analysis of A375 and A2058 cells, as well as tumor tissues, revealed that itraconazole treatment increased the expression of the pro-apoptotic protein Bax while reducing the anti-apoptotic protein Bcl-2 in both cell lines (Figures 2D, E). Additionally, itraconazole treatment for 48 h led to a significant reduction in the expression of CDK4 and Cyclin D1, key regulators of the cell cycle (Figures 2F, G). These findings suggest that itraconazole inhibits melanoma cell proliferation through apoptosis induction and G1 phase cell cycle arrest.

### 3.3 Itraconazole promotes autophagy of melanoma cells

**FIGURE 3:**
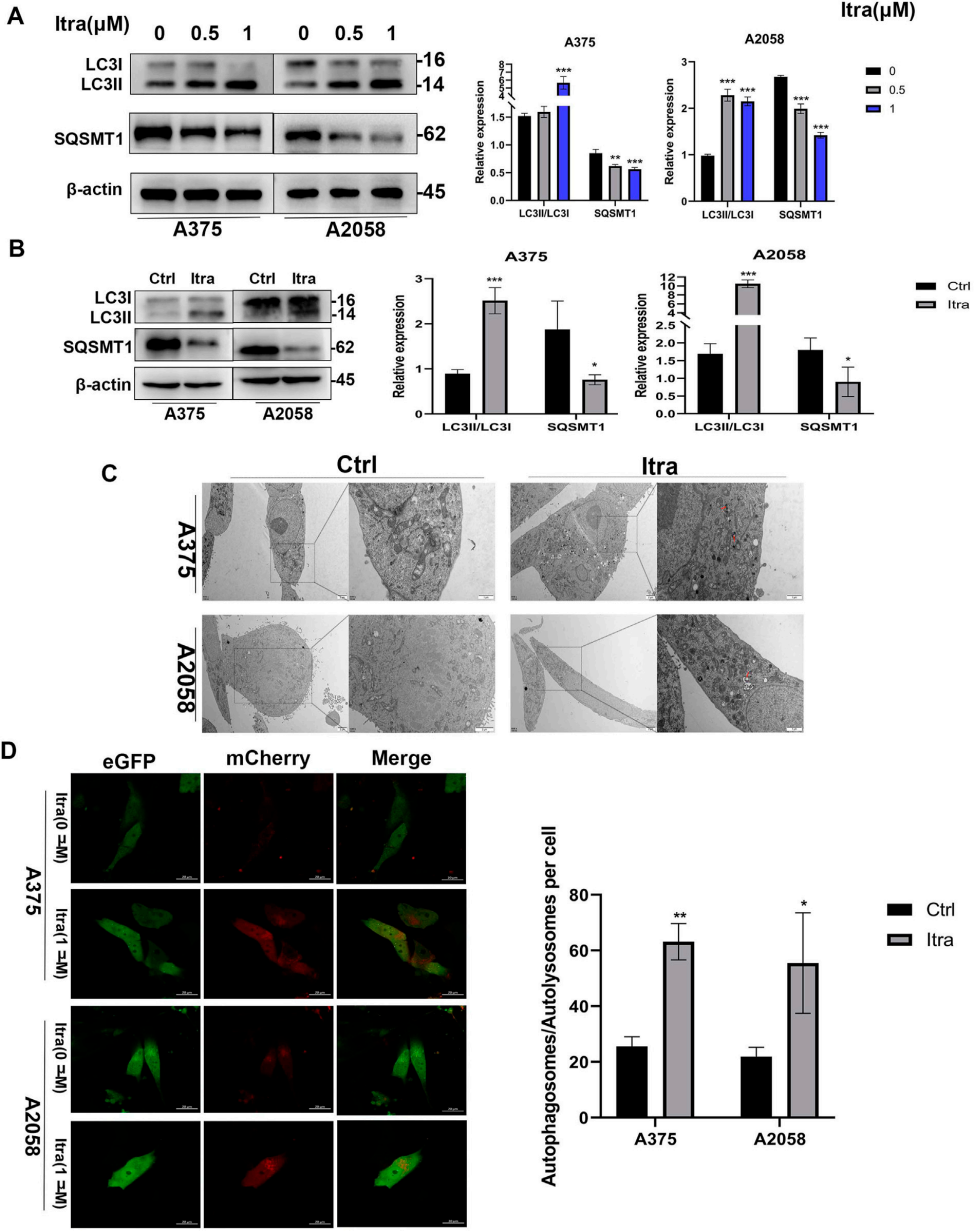
Itraconazole induces autophagy of melanoma cells. **(A, B)** LC3 and SQSTM1 protein level. **(C)** Autophagosomes was examined by transmission electron microscopy. **(D)** Cell autophagosomes and autolysosomes were observed using laser confocal microscopy. *P < 0.05; **P < 0.01; ***P < 0.001.

As emerging evidence suggests that dysregulation of autophagy contributes to tumor growth and progression (Kim et al., 2014), we examined whether itraconazole could regulate autophagy in melanoma cells. Autophagic flux, a key indicator of autophagy (Li et al., 2020), was assessed. Initially, we evaluated LC3, an autophagy marker indicative of autophagosome formation (Liu et al., 2014). In our study, the LC3-II to LC3-I ratio was significantly elevated in itraconazole-treated A375 and A2058 cells (Figure 3A). SQSTM1 (p62) was predicted as the target of Itraconazole (Supplementary Figure S1). Additionally, SQSTM1 (p62) levels were reduced, consistent with its degradation via the autophagic pathway (Sun et al., 2018). The protein expression patterns of LC3-II/LC3-I and SQSTM1 (p62) in tumor tissues from itraconazole-treated mice mirrored the *in vitro* findings (Figure 3B). To further confirm itraconazole-induced autophagy, TEM was employed to observe autophagosomes. Accumulation of autolysosomes and autophagosomes was evident in the cytoplasm of itraconazole-treated cells, but absent in controls (Figure 3C). Using a mCherry-eGFP-LC3B adenovirus vector as a marker under a laser confocal microscope, we detected an increase in red puncta representing autolysosomes following itraconazole treatment (Figure 3D). These results collectively demonstrate that itraconazole facilitates autophagy in melanoma cells.

### 3.4 Itraconazole induces apoptosis of melanoma cells through promoting autophagy

**FIGURE 4:**
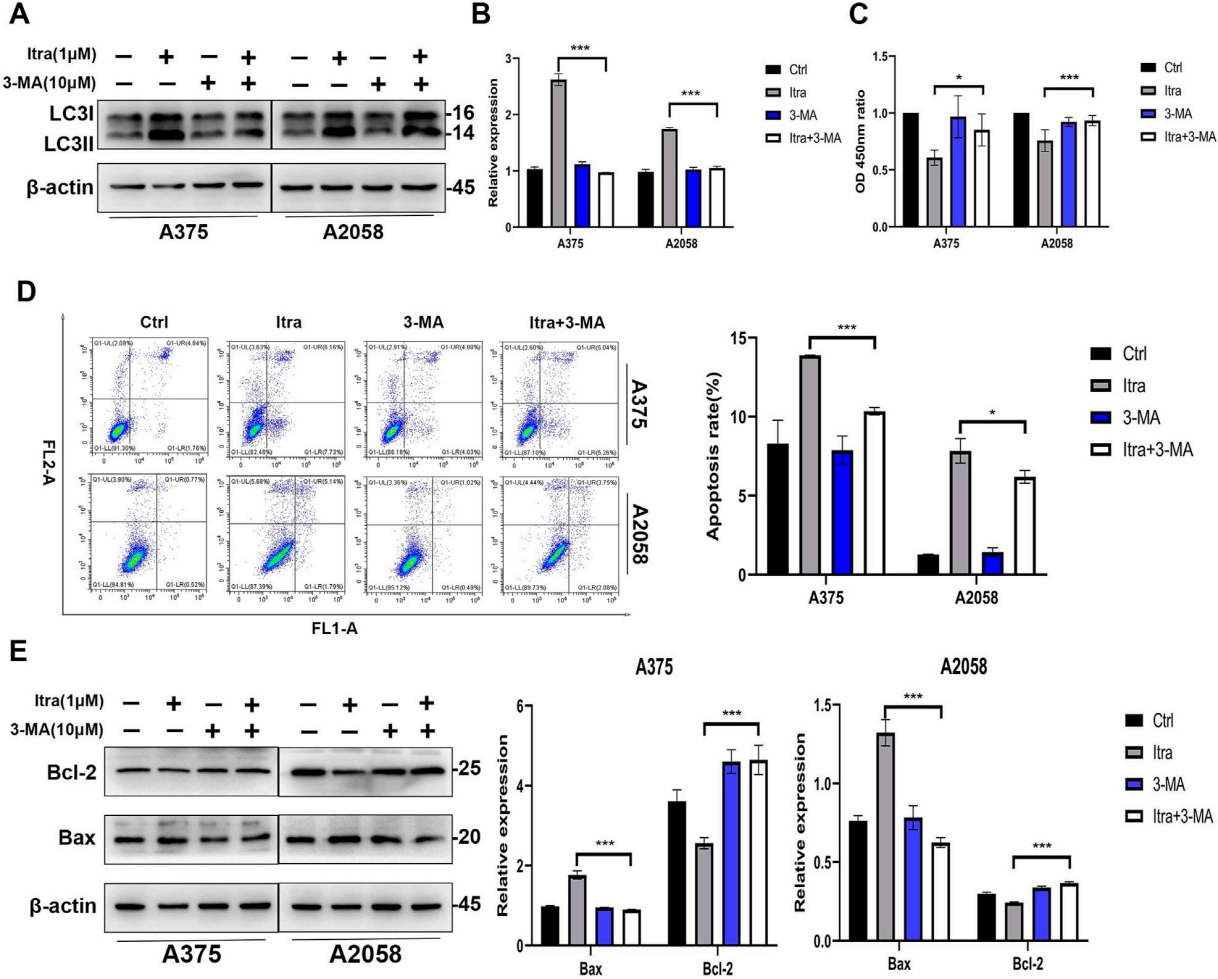
Itraconazole induces apoptosis of melanoma cells by promoting autophagy. **(A, B)** LC3 protein level in A375 and A2058 cells treated with the indicated concentrations of itraconazole. **(C)** Cell viability of A375 and A2058 cells treated with itraconazole for 48 h. **(D)** Apoptosis rate of melanoma cells was measured by flow cytometry. **(E)** Bcl-2 and Bax protein level in A375 and A2058 cells treated with itraconazole for 48 h. *P < 0.05; **P < 0.01; ***P < 0.001.

Itraconazole treatment induced autophagy in melanoma cells, as evidenced by the accumulation of autophagosomes. However, prolonged autophagic activation led to the release of pro-apoptotic signals, such as cytochrome c, resulting in apoptosis (Fan and Zong, 2013). To confirm the role of itraconazole-induced autophagy in melanoma cell apoptosis, we employed the autophagy inhibitor 3-MA. Co-treatment with itraconazole and 3-MA significantly reduced the LC3-II to LC3-I ratio compared to itraconazole treatment alone (Figures 4A, B). Moreover, the viability of melanoma cells treated with itraconazole was partially restored when combined with 3-MA, accompanied by a decrease in the apoptosis rate compared with itraconazole alone (Figures 4C, D). Consistent with these findings, the expression of the anti-apoptotic protein Bcl-2 was significantly increased, while the expression of the pro-apoptotic protein Bax was attenuated in the presence of 3-MA (Figure 4E). These results collectively demonstrate that itraconazole-induced autophagy plays a crucial role in promoting apoptosis in melanoma cells.

### 3.5 Itraconazole inhibits the Hh signaling pathway

**FIGURE 5:**
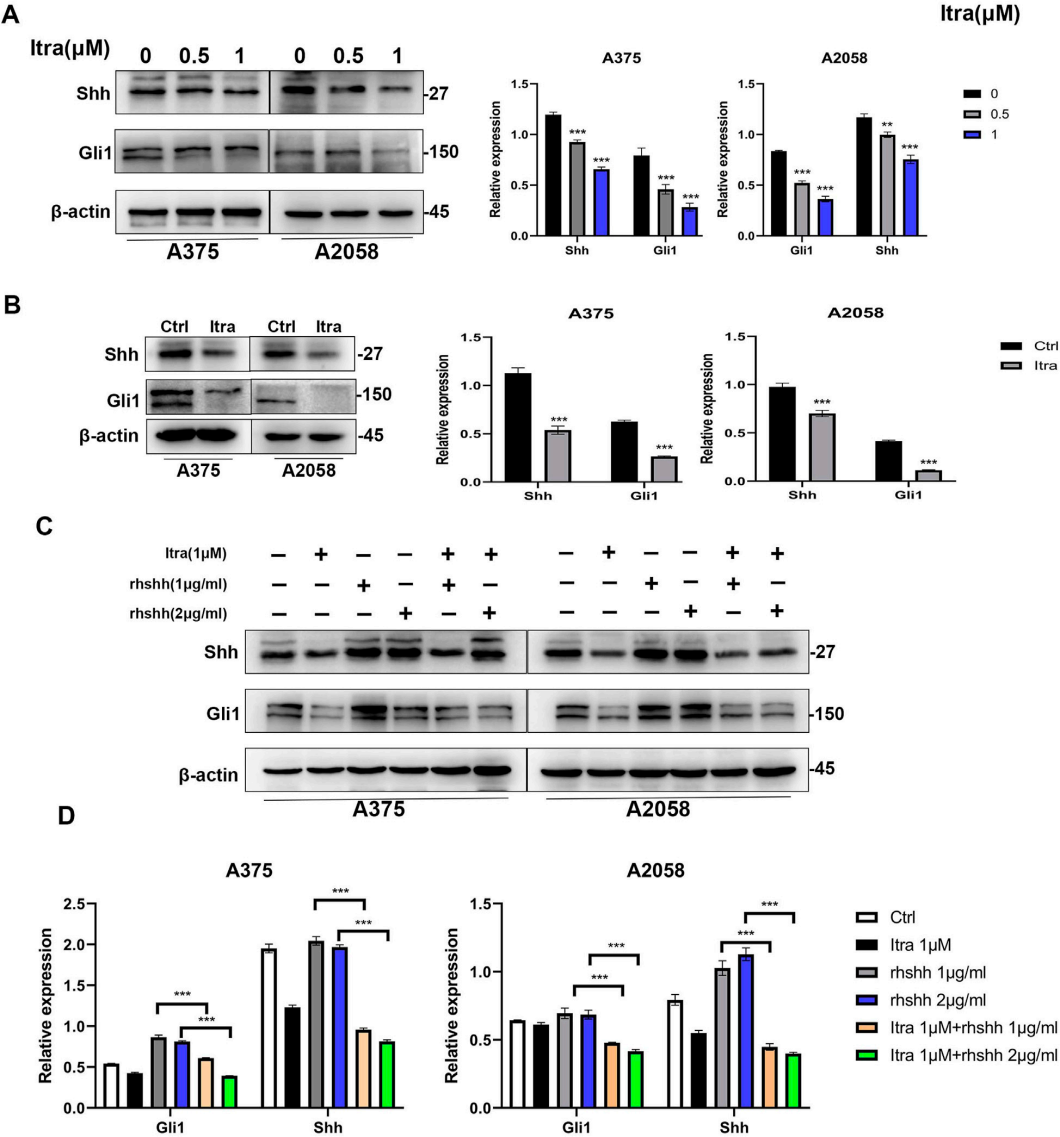
Itraconazole inhibits the Hedgehog signaling pathway. **(A, B)** Shh and Gli1 protein level. **(C, D)** Shh and Gli1 protein level in A375 and A2058 cells treated with itraconazole in the absence or presence of rhShh. *P < 0.05; **P < 0.01.

To investigate how itraconazole affects the Hedgehog (Hh) signaling pathway in melanoma cells, we analyzed key proteins involved in this pathway, including SHH, PTCH1, SMO, and the transcription factors Gli1, Gli2, and Gli3 (Moscat et al., 2016) in A375 and A2058 cell lines which were selected due to their robust response to Itraconazole and their relevance to the *in vivo* studies. SK-MEL-28 cells were not included in these specific experiments due to experimental constraints and the need for consistency with other models. Specifically, we examined the expression of SHH and Gli1 in A375 and A2058 cells. As shown in Figure 5A, itraconazole treatment led to a significant concentration-dependent reduction in the expression of these proteins. Consistently, SHH and Gli1 expression levels were also decreased in the xenograft models treated with itraconazole (Figure 5B). To further clarify itraconazole’s effect on the Hh signaling pathway, A375 and A2058 cells were treated with the pathway agonist rhShh at concentrations of 1 μg/mL and 2 μg/mL. Compared to rhShh treatment alone, co-treatment with itraconazole significantly reduced the expression of Gli1 and SHH proteins (Figures 5C, D). These findings suggest that itraconazole suppresses melanoma growth by inhibiting the Hh signaling pathway.

### 3.6 Itraconazole induces autophagy via inhibiting Hh pathway in melanoma cells

**FIGURE 6:**
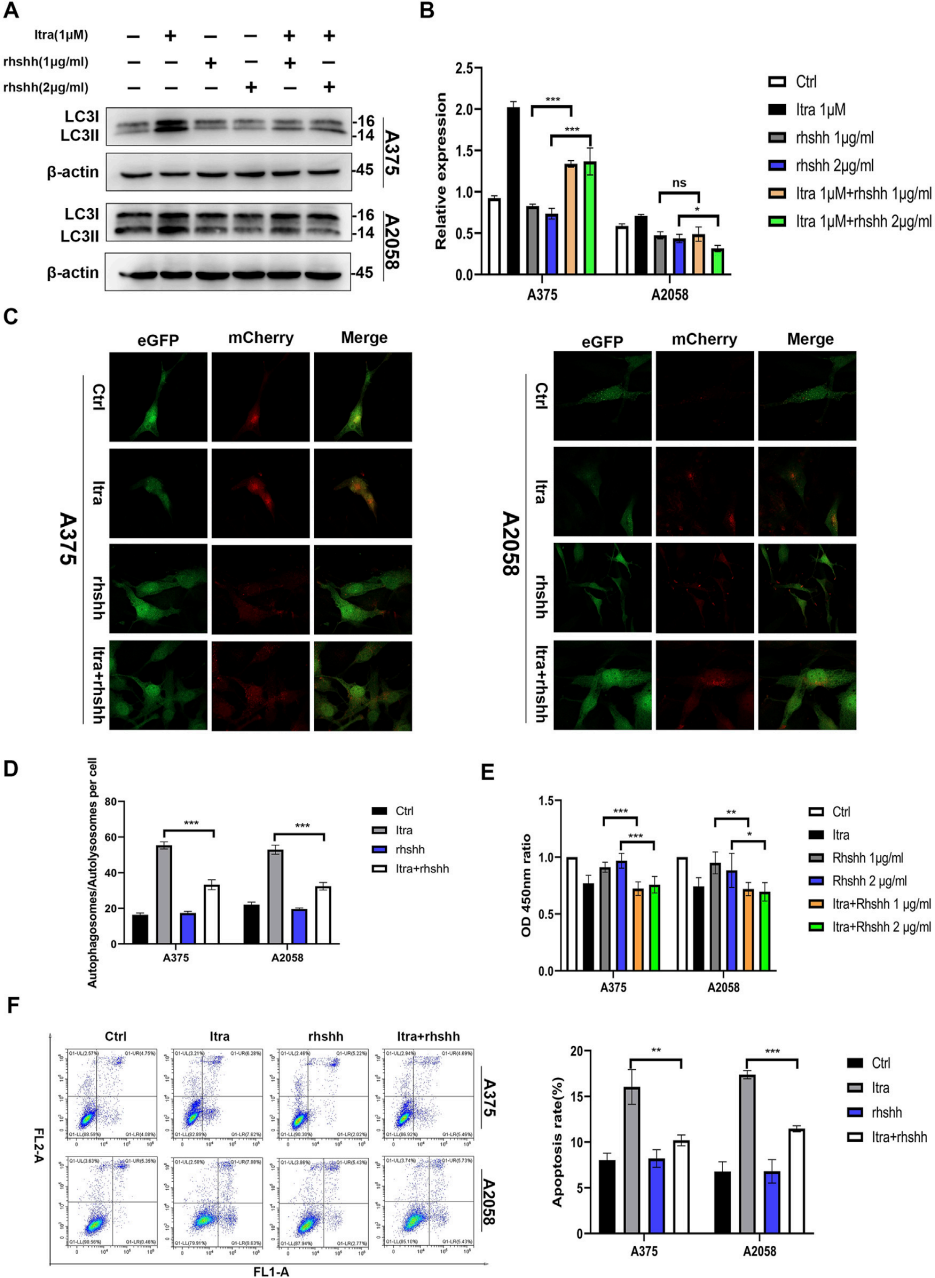
Itraconazole induces autophagy via Hh pathway inhibition in melanoma cells. **(A, B)** LC3 protein level in A375 and A2058 cells treated with itraconazole. **(C, D)** Autophagosomes and autolysosomes were observed using laser confocal microscopy. **(E)** Cell viability. **(F)** Cell apoptosis and cycle distributions were examined by flow cytometry. *P < 0.05; **P < 0.01; ns, not significant.

To investigate the regulatory mechanism by which inhibition of the Hedgehog (Hh) pathway affects itraconazole-induced autophagy in melanoma cells, we utilized the pathway agonist rhShh to activate the Hh pathway. As shown in Figures 6A, B, the proportion of LC3-II to LC3-I was significantly reduced in the itraconazole combined with rhShh groups compared to the groups treated with itraconazole alone. Similar findings were observed in A375 and A2058 melanoma cells (Figures 6C, D). Laser confocal scanning microscopy further revealed that the number of intracellular autophagosomes was markedly lower in the rhShh and itraconazole co-treatment group than in the itraconazole-only group. These results confirm that itraconazole induces autophagy in melanoma cells by suppressing the Hh signaling pathway. Additionally, data from CCK8 assays and flow cytometry (Figures 6E, F) demonstrated that recombinant rhShh could partially reverse the inhibitory effects of itraconazole on melanoma cells.

## 4 Discussion

In this study, we demonstrated that Itraconazole effectively inhibits melanoma cell proliferation and induces apoptosis in both A375 and A2058 cell lines by arresting the cell cycle in the G1 phase. Our *in vivo* studies further showed that intratumoral injection of Itraconazole significantly suppressed tumor growth in nude mice, suggesting its potential as a therapeutic agent for melanoma. The tumor suppressive effect may be caused by various factors, including genetic mutations, drug metabolism, and the activity of intracellular signaling pathways (Varjosalo and Taipale, 2008; Scales and de Sauvage, 2009). This result is consistent with previous studies that have investigated the antitumor effects of Itraconazole on melanoma (Liang et al., 2017). However, our study builds on this body of research by investigating the specific molecular mechanisms underlying Itraconazole’s effects on melanoma, with a focus on the role of autophagy and Hh signaling pathway.

Growing evidence suggests that itraconazole not only suppresses cancer via the TGF-β/SMAD2/3 signaling pathway (Chen et al., 2018), but also exerts antitumor effects on various cancer cells (endometrial cancer and gastric cancer)via Hh signaling pathway (Hu et al., 2017; Kim et al., 2010). SMO, GLI1, and SHH are prominent constitute of the Hh signaling pathway. Upon deficient Hh ligands, the PTCH receptor inhibits SMO receptor activity, and the pathway is in an inhibited state. The binding of Hh ligands to PTCH releases the inhibition by PTCH and promotes the SMO receptor to be activated, which in turn activates the Gli transcription factors, prompting them to enter the nucleus and make target genes being transcribed (Scales and de Sauvage, 2009; Wu et al., 2017; Hui and Angers, 2011). Aleksandar Sekulic et al. treated basal cell carcinoma by using Hh pathway inhibitor Vismodegib, they found objective response rates of metastatic basal cell carcinoma, and locally advanced BCC were 48.5% and 60.3%, respectively (Stecca and Ruiz i Altaba, 2010). In the study of Jorge E. Cortes et al., acute myeloid leukemia (AML) patients were administrated by Hh pathway inhibitor Glasdegib combined with low-dose cytarabine (LDAC), patients receiving the combination therapy presented obviously longer median overall survival (8.3 months) compared to those receiving LDAC alone (4.1 months) (Apalla et al., 2017). The aforementioned studies all demonstrate that inhibiting the Hh pathway has therapeutic benefits for tumors. However, researches have not examined in detail the roles of Hedgehog in melanoma. In our study, Shh and Gli1 underwent downregulation in A375 and A2058 cells receiving itraconazole treatment, proving the possible role of itraconazole in restraining melanoma growth via inhibiting Hh pathway.

Increasingly evidence has reported many signaling pathways (PI3K/AKT pathway and AMPK signaling pathway) interacting with autophagy in different cancer cell types (Heras-Sandoval et al., 2014; Hennessy et al., 2005), however, the Hh signaling pathway exhibits a superiority. According to previous studies, GLI1 in the Hh signaling pathway inhibited the expression of autophagy-related genes (ATG5 and LC3), thereby reducing autophagosome formation and autophagic flux (Stecca and Ruiz i Altaba, 2010; Guo et al., 2021). Furthermore, according to the findings by Chen activating the Hh signaling pathway markedly increased autophagic activity in ovarian cancer cells, thus facilitating cell growth and survival, while suppressing the Hh signaling pathway or autophagy greatly inhibited the cell proliferation (Pan et al., 2021). In the study by Li, activating the Hh signaling pathway promoted thyroid tumor cells to survive and develop via modulating the expression of essential autophagy genes and restricting the autophagy process. Suppressing the Hh signaling pathway can reactivate autophagy, resulting in weakened proliferation and higher apoptosis pertaining to thyroid tumor cells (Guo et al., 2021).

The Hedgehog (Hh) signaling pathway plays a critical role in the regulation of melanoma progression, contributing to cell proliferation, survival, and metastasis (Jing et al., 2023). Aberrant activation of this pathway is frequently observed in melanoma where it acts in concert with other oncogenic signaling pathways, such as MAPK and PI3K/AKT (Klein et al., 2019). Dysregulated Hh signaling in melanoma is associated with increased tumorigenicity and resistance to conventional therapies. Itraconazole has been shown to inhibit the Hh pathway by blocking Smoothened (SMO), a key receptor in this cascade, thereby preventing the activation of Gli transcription factors that regulate target genes involved in cell growth and survival. This inhibition of SMO and Gli1 by Itraconazole not only reduces melanoma cell proliferation but also induces apoptosis (Sigafoos et al., 2021). Furthermore, the crosstalk between the Hh pathway and other signaling networks, such as the PI3K/AKT and MAPK pathways, highlights the potential for Itraconazole to synergize with other therapeutic strategies, such as BRAF inhibitors or immune checkpoint inhibitors, to overcome resistance mechanisms in melanoma treatment.

In addition to its direct antitumor effects, Itraconazole may also have potential when combined with other therapeutic modalities such as radiotherapy and immunotherapy (Li et al., 2024). Autophagy, which Itraconazole modulates, plays a key role in the response of cancer cells to radiation. In certain contexts, autophagy inhibition can sensitize tumor cells to radiotherapy, potentially enhancing its efficacy. Furthermore, Itraconazole’s effects on autophagy could also modulate the tumor microenvironment, potentially enhancing the effects of immunotherapy, such as immune checkpoint inhibitors. By combining Itraconazole with these therapies, we may overcome resistance mechanisms and improve treatment outcomes in melanoma. However, further preclinical and clinical studies are needed to evaluate the safety, efficacy, and optimal combination strategies for Itraconazole with radiotherapy and immunotherapy.

Our study demonstrated that itraconazole can induce autophagy and benefit melanoma cell apoptosis through inhibiting the Hh signaling pathway. However, despite itraconazole reducing Shh and Gli1expressions, there were other autophagy-inducing factors. While we focused on the Hh signaling pathway and its interaction with autophagy, other mechanisms, such as angiogenesis, immune modulation, and extracellular matrix remodeling, may also contribute to the antitumor effects of Itraconazole. Future studies should explore these additional pathways in greater detail to provide a comprehensive understanding of Itraconazole’s full mechanism of action. Before Itraconazole can be considered for clinical use in melanoma, well-designed clinical trials are necessary to evaluate its safety, efficacy, and optimal dosing in human patients. In conclusion, our study demonstrates that Itraconazole, through its effects on the Hedgehog signaling pathway, autophagy, and apoptosis, holds significant promise as a therapeutic agent in melanoma.

## 5 Conclusion

Itraconazole induces autophagy-mediated apoptosis in melanoma cells by inhibiting Hedgehog signaling, underscoring its potential as a therapeutic option for melanoma treatment.

## Data Availability

The original contributions presented in the study are included in the article/Supplementary Material, further inquiries can be directed to the corresponding authors.
