# Supplementary figures and images for: Itraconazole promotes melanoma cells apoptosis via inhibiting hedgehog signaling pathway-mediated autophagy
Source: Front Pharmacol. 2025 Jan 23;16:1545243. doi: 10.3389/fphar.2025.1545243 (PMC11798931; doi:10.3389/fphar.2025.1545243)

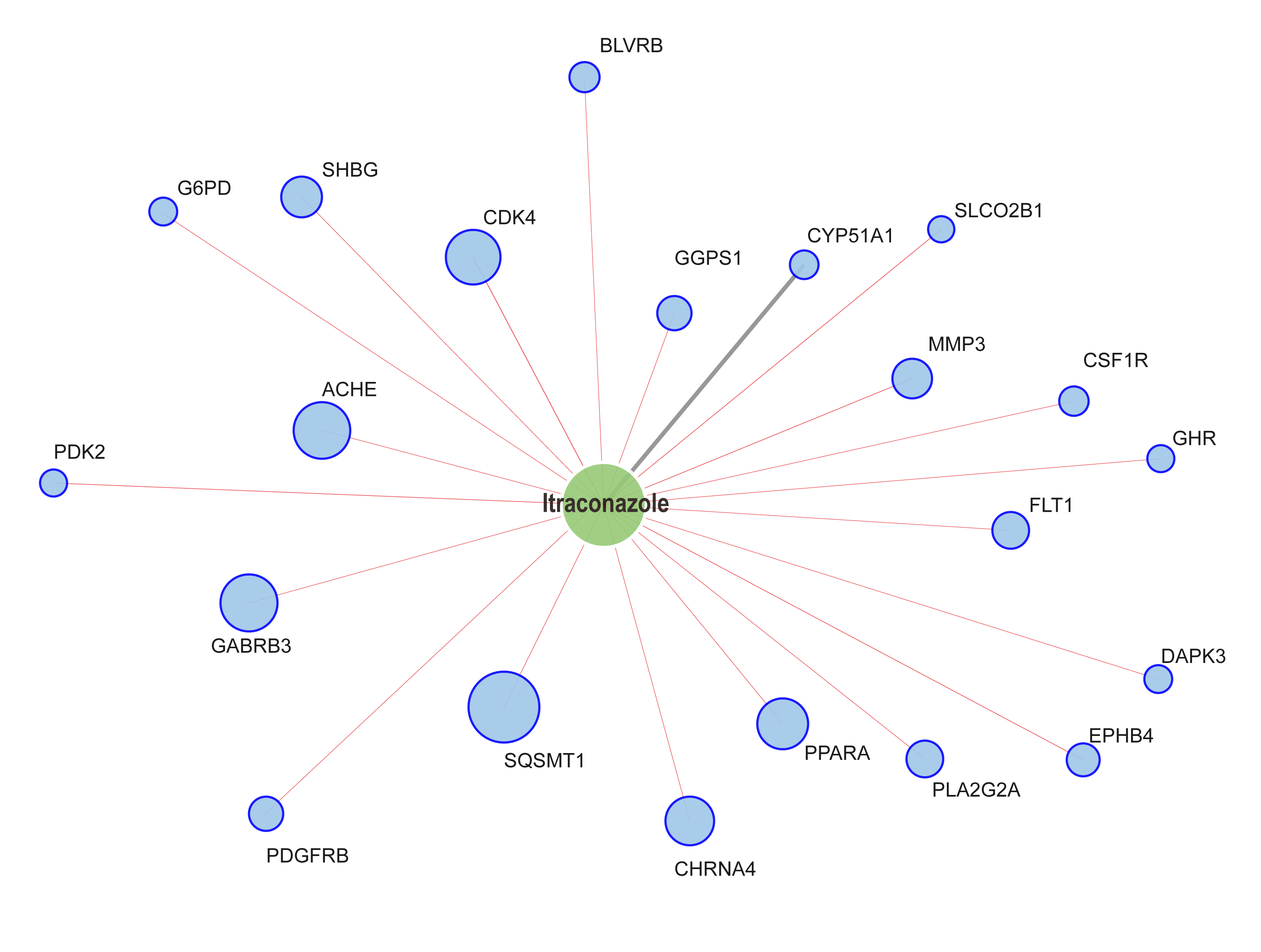

Supplement: Supplementary file 1 [file Image1.tif]
